# Variable Neuronal Participation in Stereotypic Motor Programs

**DOI:** 10.1371/journal.pone.0040579

**Published:** 2012-07-16

**Authors:** Evan S. Hill, Sunil K. Vasireddi, Angela M. Bruno, Jean Wang, William N. Frost

**Affiliations:** 1 Department of Cell Biology and Anatomy, The Chicago Medical School, Rosalind Franklin University of Medicine and Science, North Chicago, Illinois, United States of America; 2 Department of Neuroscience, The Chicago Medical School, Rosalind Franklin University of Medicine and Science, North Chicago, Illinois, United States of America; Georgia State University, United States of America

## Abstract

To what extent are motor networks underlying rhythmic behaviors rigidly hard-wired versus fluid and dynamic entities? Do the members of motor networks change from moment-to-moment or from motor program episode-to-episode? These are questions that can only be addressed in systems where it is possible to monitor the spiking activity of networks of neurons during the production of motor programs. We used large-scale voltage-sensitive dye (VSD) imaging followed by Independent Component Analysis spike-sorting to examine the extent to which the neuronal network underlying the escape swim behavior of *Tritonia diomedea* is hard-wired versus fluid from a moment-to-moment perspective. We found that while most neurons were dedicated to the swim network, a small but significant proportion of neurons participated in a surprisingly variable manner. These neurons joined the swim motor program late, left early, burst only on some cycles or skipped cycles of the motor program. We confirmed that this variable neuronal participation was not due to effects of the VSD by finding such neurons with intracellular recording in dye-free saline. Further, these neurons markedly varied their level of participation in the network from swim episode-to-episode. The generality of such unreliably bursting neurons was confirmed by their presence in the rhythmic escape networks of two other molluscan species, *Tritonia festiva* and *Aplysia californica*. Our observations support a view that neuronal networks, even those underlying rhythmic and stereotyped motor programs, may be more variable in structure than widely appreciated.

## Introduction

Traditionally, rhythmic networks underlying various motor behaviors have been depicted in the literature by circuit diagrams, giving the impression that neurons always participate reliably in networks. While multifunctional neurons that participate in multiple distinct motor networks have been described in many species [1]–[9], examples of neurons that vary their level of participation in single motor networks are less common.

Any given rhythmic behavior could be produced by a fixed number of “hard-wired” neurons [10], [11], shifting assemblies of neurons, or a combination of the two. To address this issue, the activity of neuronal populations must be monitored while motor programs are running. This is challenging in vertebrate preparations, where motor networks underlying rhythmic behaviors are comprised of thousands of neurons that are often distributed along the spinal cord [12]. Fortunately, certain invertebrate motor networks are comprised of comparatively few, large-diameter neurons, located on the periphery of central ganglia, making it possible to optically record the simultaneous activity of neuronal populations.

**Figure 1:**
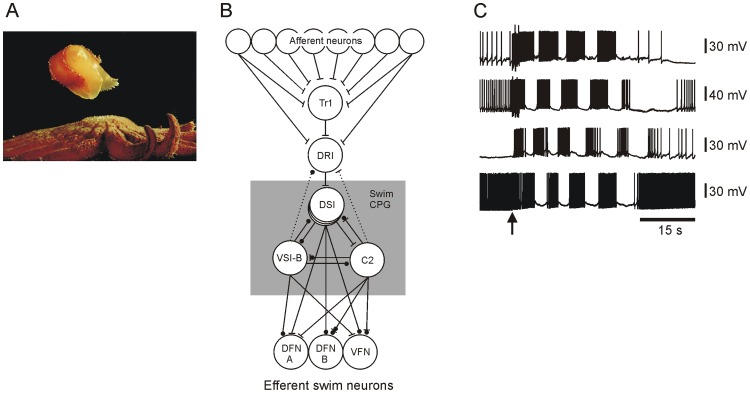
*Tritonia diomedea* escape swim behavior, the neuronal network underlying the escape swim motor program (SMP), and typical firing activity of four flexion neurons during the SMP. **A** Upon skin contact with its sea star predators, the marine nudibranch mollusk *T. diomedea* launches a rhythmic escape swim motor program consisting of several cycles of alternating ventral and dorsal whole-body flexions. In this photo the animal is at the point of full ventral flexion. **B** Previous studies have described a detailed neuronal network mediating this motor program, consisting of afferent neurons (s-cells), trigger neurons (TRI), swim command neurons (DRI), central pattern generator neurons (C2, DSI, VSI-B), and motor efferent neurons (DFN-A, DFN-B, VFN). The known afferent neurons and interneurons are located in the fused cerebral-pleural ganglia, while the flexion neurons are located in the pedal ganglia. Bars represent excitatory connections and circles represent inhibitory connections. **C** Example of a typical escape SMP - intracellular recording from four flexion neurons. These flexion neurons all fired bursts of action potentials on every cycle of the SMP. Arrow –10 V, 10 Hz, 2 s stimulus to pedal nerve 3 (PdN 3).

When the marine mollusk *Tritonia diomedea* perceives a suitably aversive skin stimulus it launches a rhythmic escape swim response consisting of a series of alternating ventral and dorsal whole-body flexions that propel it to safety (**Fig. 1A**). *T. diomedea* has been an attractive model preparation for network studies, in part because the rhythmic motor program underlying this behavior can be studied in isolated brain preparations [13]. It has been a useful model for various neurobiological topics, including learning, modulation, pattern generation and prepulse inhibition [14]–[23].

Most prior studies of the *T. diomedea* escape swim network employed sharp electrode recordings from three to four neurons. Here we used large-scale fast voltage-sensitive dye (VSD) imaging followed by Independent Component Analysis (ICA) spike-sorting to simultaneously monitor the activity of populations of *T. diomedea* swim network neurons, providing a greatly expanded view of network function. Our optical data revealed that while the majority of neurons that burst rhythmically during the swim motor program (SMP) fired action potentials on every cycle of every motor program, a small but significant number of neurons participated in the network variably from cycle-to-cycle and from episode-to-episode. The possible generality of this result was confirmed by the presence of similar variably-committed neurons in the escape locomotion networks of two additional molluscan species, *Tritonia festiva* and *Aplysia californica*. Our results support a view of neuronal networks, even those mediating highly stereotypic behaviors, as being composed of shifting coalitions of neurons, such that network structure can change in a highly fluid fashion over time.

## Results

Publications from sharp electrode studies of rhythmic motor networks often include a circuit diagram of the motor network under study (see **Fig. 1B** for the *T. diomedea* escape swim network), and the accompanying electrophysiology traces nearly always show network neurons reliably firing action potentials during all cycles of the motor program (**Fig. 1C** **;** [15], [21], [24], [25]). Fast VSD imaging combined with ICA allows us to monitor the spiking activity of a greatly increased number of neurons (up to ∼120), with single-cell and sub-millisecond resolution, giving a much larger view of nervous system activity during the *T. diomedea* escape SMP. In this study, all independent component traces that burst in phase with the swim rhythm were deemed swim network neurons.

**Figure 2:**
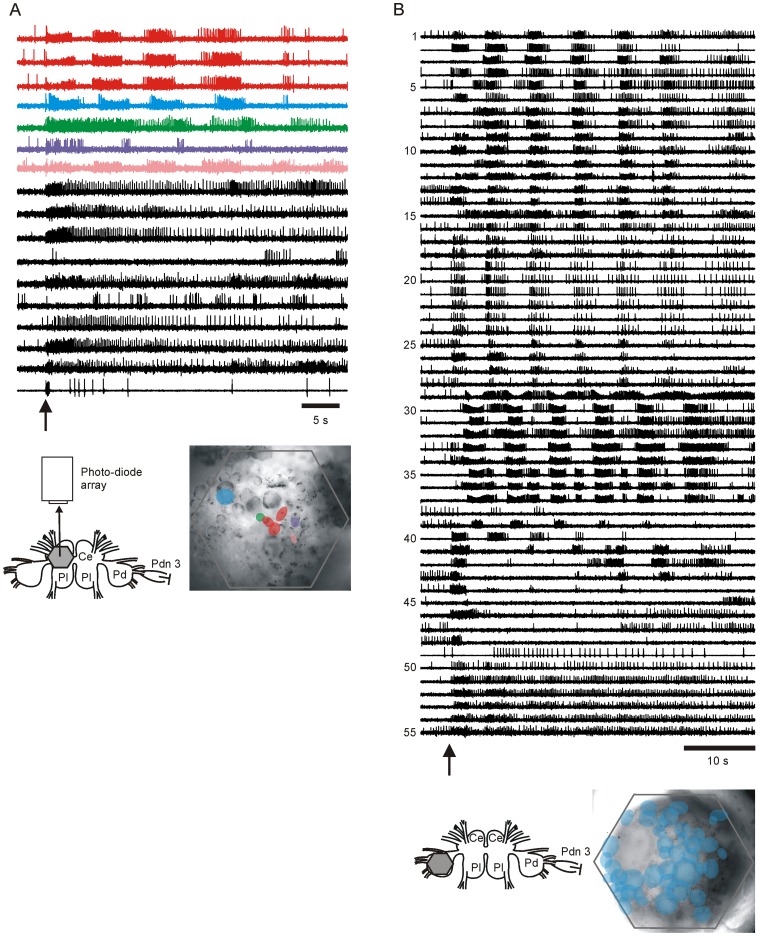
VSD imaging in the cerebral and pedal ganglia revealed the presence of known types of swim network neurons, as well as unknown types, including unreliably bursting neurons. **A** Imaging of the dorsal cerebral ganglion. Prior sharp electrode studies have identified C2, the DSIs and VSI-A as swim CPG neurons located on the dorsal surface of the cerebral ganglion. The bursting activity and map locations of some of the neurons present in optical recordings of the dorsal cerebral ganglion correspond to the known bursting activity during the SMP and spatial locations of these CPG neurons (C2 - shown in blue, the DSIs - shown in red, and VSI-A - shown in green). Further, the activity of other unknown bursting neurons was present in the optical recording (purple and pink traces and maps), along with other neurons that were either inhibited or tonically active during the SMP. Additionally, some neurons fired bursts on some but not all cycles of the SMP (top three black traces). Inset - experimental set-up: a 464-element photo-diode array was used to image the activity of neurons in the dorsal cerebral ganglion in response to a stimulus given to the contralateral PdN 3. The locations of reliably bursting neurons are shown superimposed over an image of the cerebral ganglion (the colors of the cells match the color of the traces). **B** Imaging in the dorsal pedal ganglion revealed the presence of several types of known efferent flexion neurons including type A and B dorsal flexion neurons (DFN-A and DFN-B, lines 1 to 16), ventral flexion neurons (VFNs, lines 29 to 34) and a neuron type that fires two bursts per cycle instead of one (Type III neurons, lines 35 to 37). Neurons that fired weakly in the dorsal phase of the SMP were also present in the recording (lines 17 to 28), as were neurons that were inhibited or fired tonically during the SMP (lines 45 to 49). Finally, some neurons fired on some but not all cycles of the SMP (lines 38 to 44). Inset – the imaging location is depicted. The locations of all the bursting neurons are shown in blue superimposed over an image of the dorsal pedal ganglion. Ce – cerebral ganglion, Pl – pleural ganglion, Pd – pedal ganglion; arrows - 10 V, 10 Hz, 2 s stimulus to contralateral PdN 3.

Our initial experiments focused on testing whether we could optically detect the known *T. diomedea* swim network neurons that have previously been described from sharp electrode studies. *Dorsal Cerebral Ganglion*. Several members of the *Tritonia* swim CPG are located on the dorsal surface of the cerebral ganglion. These include Cerebral Neuron 2, (C2), the 3 Dorsal Swim Interneurons (DSIs), and the Type-A Ventral Swim Interneuron (VSI-A). Neurons firing in the characteristic manner and at the location of all five of these neurons appeared in our optical recordings of nerve-evoked SMPs (**Fig. 2A**). Imaging also revealed the presence of some bursting neurons that have not been previously described (**Fig. 2A**, purple and pink traces and maps), including some neurons that skipped cycles of the motor program (**Fig. 2A**, top three black traces). *Dorsal Pedal Ganglion*. Next, optical recordings of the dorsal pedal ganglion, the location of most of the known efferent neurons for the swim motor network [26], [27], typically recorded the action potentials of several dozen neurons. **Figure** **2B** shows the simultaneously imaged activity of 55 dorsal pedal ganglion neurons, most of whose activity corresponded to the previously described categories of swim flexion neurons. These included the Type-A and Type-B Dorsal Flexion Neurons (DFN-A, DFN-B), which burst in the dorsal flexion phase of the swim, the Ventral Flexion Neurons (VFN), which burst in the ventral flexion phase, and Type III neurons, which burst in both phases [26]. The DFN and VFN neurons were also detected in prior pedal ganglion optical recordings from another laboratory [28]. The diodes contributing to the independent components returned by ICA were used to determine the ganglion location of each detected neuron [28], [29]. The rhythmically bursting neurons were distributed widely across the dorsal pedal ganglion (**Fig. 2B**). The optical recordings also detected neurons that were inhibited, and others that were tonically active during the SMP, along with some neurons that fired on some but not all cycles of the SMP (**Fig. 2B** **,** lines 38 to 44).

**Figure 3:**
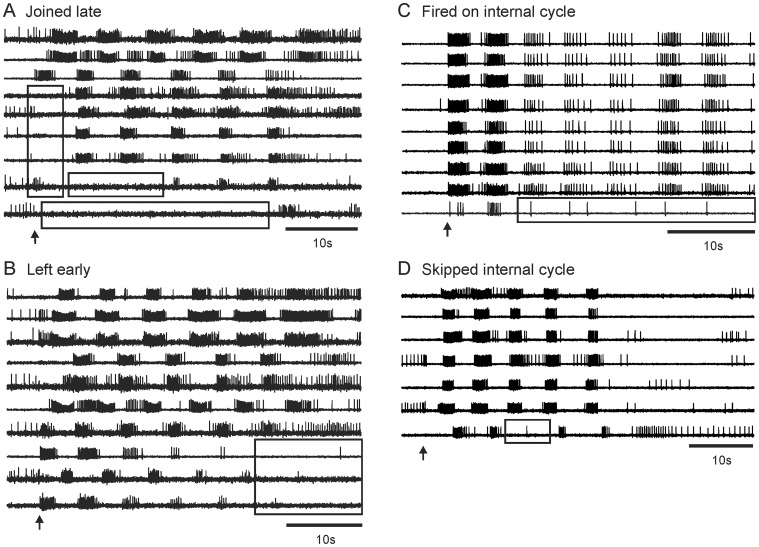
Large-scale optical imaging revealed four classes of neurons that participate unreliably in the *T. diomedea* escape SMP. **A** Optical recording from the dorsal pedal ganglion, showing neurons that joined the motor program late. **B** Example from the dorsal pedal ganglion showing neurons that left the motor program early. **C** Example from the ventral pedal ganglion of a neuron that participated in only one cycle of the motor program. **D** Example from the dorsal pedal ganglion of a neuron that skipped an internal cycle of the motor program.

**Figure 4:**
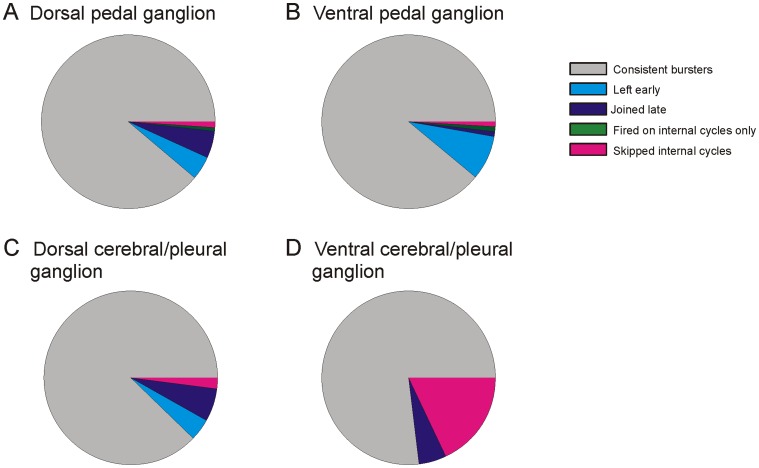
Distribution of the types of unreliably bursting neurons in the pedal, cerebral and pleural ganglia of *T. diomedea*. **A** In the dorsal pedal ganglion 11.4% of the neurons that burst during the SMP did so in an unreliable fashion (52 out of 458 bursting neurons in 19 motor programs in 19 preparations). Of the unreliable bursters, 38.4% left early, 44.2% joined late, 9.6% skipped internal cycles and 5.8% fired on internal cycles only. **B** In the ventral pedal ganglion 11.1% of the bursting neurons skipped cycles (12 out of 108 bursting neurons in 6 motor programs in 6 preparations). Of the unreliable bursters, 75% left early, 8.3% joined late, 8.3% skipped internal cycles and 8.3% fired on internal cycles only. **C** In the dorsal cerebral/pleural ganglia 12.2% of the bursting neurons skipped cycles (6 out of 49 bursting neurons in 12 motor programs in 12 preparations). Of the unreliable bursters, 33.3% left early, 50% joined late, and 16.6% skipped internal cycles. **D** In the ventral cerebral/pleural ganglia 23.1% of the bursting neurons skipped cycles (9 out of 39 bursting neurons in 7 motor programs in 7 preparations). Of the unreliable bursters, 22.2% joined late, and 77.8% skipped internal cycles.

While most neurons that participated in the SMP fired consistently on every cycle, some participated unreliably, bursting during some cycles but not others (see methods for a detailed description of how neurons were identified as being semi-committed to the SMP). Neurons skipped one or more cycles of the escape SMP in 29 out of 45 optical recordings of *T. diomedea* central ganglia in 43 preparations (in two preparations both the dorsal pedal and dorsal cerebral/pleural ganglia were imaged). These neurons, referred to here as “semi-committed” or “unreliably bursting” neurons, were of four types: those that 1) joined the motor program after it was underway, 2) left before it was finished, 3) skipped internal cycles, or 4) burst on one or a few internal cycles of the motor program (**Fig. 3**). While most of our recordings focused on the dorsal pedal ganglia, such semi-committed neurons were also observed in the ventral pedal ganglia and in the ventral and dorsal cerebral and pleural ganglia, indicating this to be a widespread phenomenon in the *T. diomedea* nervous system (**Fig. 4**). A summary of all optically recorded fully- and semi-committed swim network neurons in *T. diomedea* ganglia is shown in Table 1. Our location maps of the optically recorded neurons showed that both fully and semi-committed participants in the SMP were heterogeneously distributed throughout the pedal ganglion. Of the neurons recorded on the dorsal sides of the ganglia, semi-committed neurons were roughly equally divided with respect to whether they burst in the dorsal or ventral phase of the SMP (42% of semi-committed neurons in the dorsal pedal ganglion burst in the dorsal phase of the SMP, as did 57% of semi-committed neurons in the dorsal cerebral/pleural ganglia). In contrast, the unreliably bursting neurons located on the ventral sides of the ganglia mainly burst during the ventral phase of the SMP (92% and 90% for the ventral pedal and ventral cerebral/pleural ganglia, respectively). However, it should be noted that a great majority of rhythmically active neurons that were located on the ventral sides of the ganglia burst during the ventral phase of the SMP.

**Table 1:** Summary of optical recording of reliably and unreliably bursting swim network neurons in *T. diomedea* central ganglia.

	Bursting neuronsfully committedto the SMP	Bursting neurons that joined late	Bursting neurons that left early	Bursting neurons that fired on internal cycles only	Bursting neurons that skipped internal cycle(s)	Total number of bursting neurons
Dorsal Pedal (n = 19recordings in 19 animals)	406	23	20	3	5	457
Ventral Pedal (n = 6recordings in 6 animals)	108	1	9	1	1	120
Dorsal cerebral/pleural (n = 12 recordings in 12 animals)	43	3	2	0	1	49
Ventral cerebral/pleural(n = 7recordings in 7 animals)	30	2	0	0	7	39
Totals	587	29	31	4	14	665

Each row shows the number of optical recordings performed in each ganglion and the number of bursting neurons in each ganglion that were either fully committed to the swim motor program or were semi-committed to it (joined late, left early, fired on internal cycles, or skipped internal cycles), as well as the total number of bursting neurons that were recorded in each ganglion.

**Figure 5:**
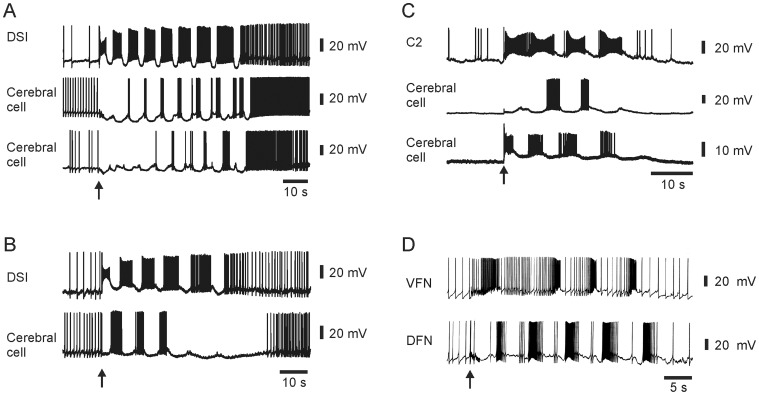
Intracellular recording with sharp electrodes revealed the presence of all four types of unreliably bursting neurons in VSD-free saline in *T. diomedea*. **A** Intracellular recording from a DSI and two additional cerebral ganglion neurons during a SMP showed that the latter two cerebral ganglion neurons skipped several of the early swim cycles before firing vigorously on the last few cycles. **B** Intracellular recording from a DSI and an additional cerebral ganglion neuron during an SMP showed that the latter neuron fired on the early swim cycles, but did not fire action potentials on the last two cycles. **C** Intracellular recording from C2 and two additional cerebral ganglion neurons during an SMP showed that the middle cerebral neuron fired action potentials only on the middle two cycles of the four cycle motor program. **D** Intracellular recording from a VFN and a DFN during an SMP showed that the VFN skipped the second cycle of the five cycle motor program.

Since it has been reported that some VSDs can affect neuronal excitability and can suppress network activity [30] or can alter the phase of rhythmically bursting neurons [31] we were concerned that the unreliable neuronal participation we observed might have been caused by application of the VSD RH-155. Evidence against this possibility comes from a survey for unreliably bursting neurons in *T. diomedea* motor program recordings made with sharp electrode intracellular recording in dye-free saline. In such conditions, we found 63 neurons in 45 preparations that participated unreliably in the SMP. Such neurons were found in the cerebral, pleural and pedal ganglia. The same four categories of semi-committed participation in the swim rhythm that we observed with the optical recording data were found, including neurons that joined the rhythm late, left early, fired on only internal cycles, or skipped internal cycles (**Fig. 5**). These findings of unreliably bursting neurons both in the presence and absence of the VSD lead us to conclude that they are a natural feature of the *T. diomedea* swim network. Additionally, ICA spike-sorting can be discounted as the source of the unreliable bursting behavior that we observed since neurons were observed to burst in this manner in the raw, pre-ICA optical data (data not shown). Furthermore, we recently validated the accuracy of ICA with simultaneous intracellular and optical recording in *T. diomedea* and *A. californica*: in each case an independent component matched the intracellular data spike-for-spike [29].

**Figure 6:**
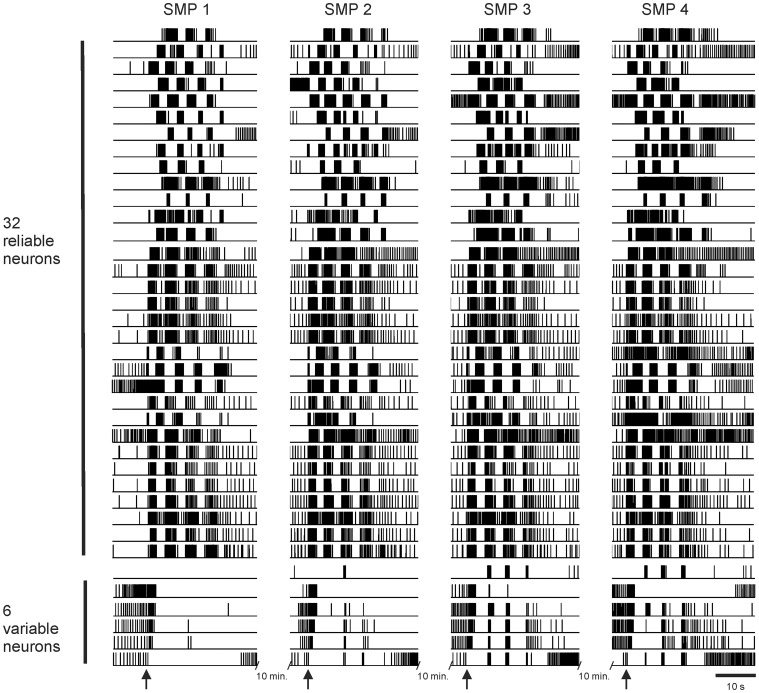
Unreliably bursting neurons varied their bursting pattern from escape swim episode to episode. Four escape swim episodes elicited by identical stimuli delivered 10 minutes apart were imaged in the dorsal pedal ganglion of *T. diomedea*. The individual optical files were concatenated prior to performing ICA in order to track the activity of individual neurons from episode to episode. For clarity, binary versions (spikes  =  ones, all other time points  =  zeros) of the component traces are shown. While 32 of 38 neurons burst in a consistent manner from swim episode to swim episode, 6 out of 38 neurons greatly varied their level of participation in the swim network. For example, some of these variably participating neurons were not rhythmically active during some swim episodes but then participated in some or all cycles of other swim episodes. Similar results were observed in seven preparations. Arrows – stimuli to pedal nerve 3 (10 V, 10 Hz, 2 s).

The variably participating neurons could be hardwired to fire in exactly the same way in each instance of the SMP (e.g. always skip the third cycle), or they could vary their level of commitment to the SMP from episode to episode. The latter possibility is especially intriguing, because it raises the scenario of neurons wandering in and out of the motor program in a variable fashion from swim episode to episode. To distinguish between these two possibilities, we elicited four SMPs ten minutes apart using identical stimuli, and imaged neuronal responses in the *T. diomedea* dorsal pedal ganglion. In these experiments, the identical region of the pedal ganglion was imaged for each SMP. The separate optical data files were concatenated in MATLAB before performing ICA, making it possible to follow the firing behavior of each recorded neuron across the swim episodes. We found that from swim episode-to-episode, while many neurons were very consistent in their bursting pattern, some neurons varied their bursting pattern greatly, transitioning from not bursting at all in one swim episode to bursting on only some cycles in another episode (semi-committed to the network), to bursting on every cycle in other swim episodes (**Fig. 6**). Similar results were obtained in seven preparations. These results reveal that the swim network is comprised of a group of neurons that participate reliably on all cycles of every motor program, and another group of neurons that move in and out of the swim network from cycle-to-cycle and from episode-to-episode.

**Figure 7:**
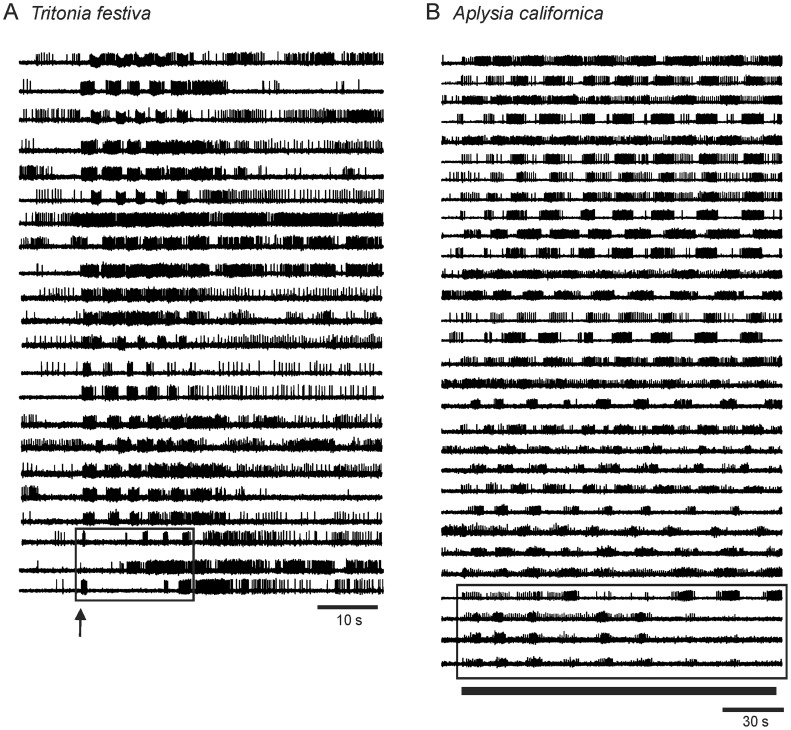
Large-scale optical imaging revealed that some neurons participate unreliably in the *T. festiva* escape SMP and the *A. californica* escape crawling motor program. **A** Optical recording of a *T. festiva* escape SMP in the dorsal pedal ganglion revealed that while most neurons fired bursts of action potentials on every cycle of the motor program, some neurons joined the motor program late (box). The arrow indicates the time of the stimulus to pedal nerve 3 (10 V, 10 Hz, 1 s). **B** Optical recording of an *A. californica* escape locomotion motor program in the dorsal pedal ganglion elicited by a 10 V, 1 Hz, 155 s stimulus to pedal nerve 9 (bar) revealed that while most neurons fired bursts of action potentials on every cycle of the motor program, a few neurons skipped some cycles (box).

Because such moment-to-moment variability in participation by neurons in a stereotypic rhythmic motor program was surprising, we investigated its generality by testing whether similar semi-committed neurons were present in the escape locomotion networks of two additional gastropod species, *Tritonia festiva* and *Aplysia californica*. We observed that some neurons in the pedal ganglion of *T. festiva* participated in some but not all cycles of the escape SMP (**Fig. 7A**, similar results were observed in three preparations). Similarly, in 14 out of 16 optical recordings (in six preparations) of the *A. californica* pedal ganglion rhythmic escape crawling motor program we observed that some neurons failed to fire on every cycle of the motor program (**Fig. 7B**). These findings suggest that such unreliably bursting neurons may be a general feature of rhythmic motor networks, but become readily evident only when the activity of large numbers of neurons are monitored simultaneously.

## Discussion

**Figure 8:**
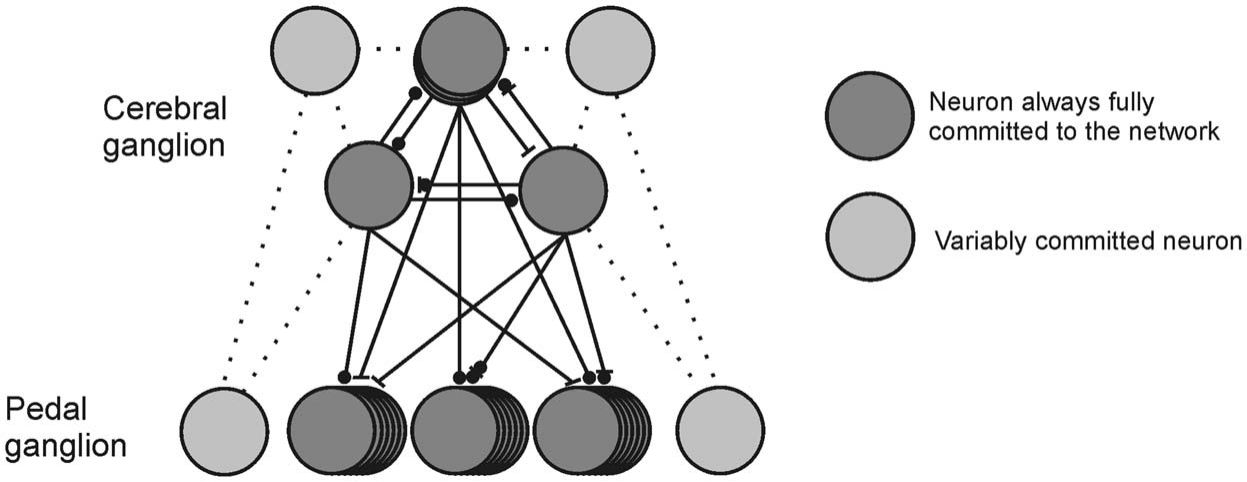
Schematic diagram depicting the *T. diomedea* swim network. Our present data suggest that the *T. diomedea* swim network is comprised of a core of neurons that are always fully committed to the network (dark grey circles), and an additional pool of neurons that vary their level of commitment to the network (light grey circles). Bars represent excitatory connections and circles represent inhibitory connections. The dotted lines represent possible synaptic connections between fully and variably committed neurons, as well as possible synaptic connections between variably committed neurons.

Our large-scale optical recording data revealed that while most of the neurons that comprise the *T. diomedea* escape swim motor network fire action potentials on every cycle of every episode of the motor program, a subset of neurons participate in the network in an unreliable or variable manner. Rather than being rigidly hard-wired and fixed in size, our findings demonstrate that the swim network underlying the highly stereotypic escape swim behavior of *T. diomedea* is comprised of a dedicated core of neurons and a dynamic, constantly changing assembly of neurons that vary their level of participation in the motor network from cycle-to-cycle and from episode-to-episode (**Fig. 8**). Our present findings add to a growing appreciation that neuronal networks may be far more flexible on a moment-to-moment basis than was recognized only a few years ago. While reminiscent of prior examples of variable neuronal participation in networks, our findings are unique in that they demonstrate that even motor networks underlying highly stereotypic behaviors can exhibit dynamic moment-to-moment flexibility in network structure.

While there are several published examples of variable participation by neurons in rhythmic motor programs, these tend to be associated with behaviors that are themselves variable in nature. The neuronal network underlying feeding behavior of *A. californica* has been shown to be highly variable in terms of cycle period, program duration and inter-program interval [32]. This network variability reflects the highly variable nature of the *A. californica* feeding behavior, where a trial-and-error feeding strategy that produces a wide range of movements may be optimal for responding to rapidly changing environmental conditions [32]. Next, in the motor program that produces scratching in turtles, some cycles of the scratching rhythm are skipped by nearly all the neurons of the network, resulting in behavioral deletions or skips [33]. These findings in *A. californica* feeding and turtle scratching are quite different conceptually from our findings in *T. diomedea*, which involve a reliable, stereotyped behavior mediated in part by group of variably participating neurons.

A similar core and non-core network structure has recently been described in the mammalian cortex. Cortical ensembles, corresponding to UP states, arising either spontaneously or in response to thalamic stimulation, were found to be comprised of a core of neurons that were always activated, and a changing complement of non-core cells that varied in its participation during each UP state [34]. Although these UP states are not rhythmic and stereotyped in nature and the exact role that they play in cortical processing is unknown, the similarities with our findings in invertebrate motor networks suggest that this type of core and non-core arrangement may be a general feature of neuronal ensembles.

Neurons that burst on only some cycles of rhythmic motor programs have been observed in the larval zebrafish spinal network, however such neurons were found to be essentially hard-wired to always fire during certain cycles of the motor program. For example, one group of zebrafish spinal interneurons fires bursts of action potentials at the beginning of the escape motor program, whereas other interneurons are recruited in to fire bursts at the end of the motor program, while the first group of interneurons is inhibited [35]. While this finding may at first seem similar to our findings in *T. diomedea*, the key difference is that these zebrafish spinal interneurons appear to be hard-wired to always burst at the beginning or at the end of the escape motor program [35]. This is in contrast to the unreliably bursting neurons in the *T. diomedea* swim network, which vary their participation from swim cycle-to-cycle, and from episode-to-episode (**Fig. 8**).

Neurons that participate only during certain phases of non-rhythmic motor patterns have also been reported in the literature. For example, songbird HVC neurons fire bursts of spikes at precise times during a song, such that each neuron only contributes to a small segment of the whole song. With repeated performances of the song, each neuron bursts at precisely the same time in the song sequence [36]. These findings are in contrast to our findings that repeated episodes of the very stereotyped *T. diomedea* escape swim may be produced by varying assemblies of neurons.

Variably participating neurons have also been reported to underlie complex, volitional movements. For example, some monkey motor cortex neurons fire spikes during some episodes of arm reaching and not during others [37]. However, monkey arm reaching is a complex, volitional movement in three dimensions with proprioceptive feedback. Thus the observed variability may reflect the production of slightly different arm movements by slightly different ensembles of neurons from trial to trial. The variable neuronal participation we observed in *T. diomedea* occurs in response to identical stimuli applied to deafferented nervous systems generating a highly stereotyped escape swim behavior.

We observed that some *T. diomedea* swim network neurons varied their level of commitment from being non-bursting, to bursting unreliably, to bursting on every cycle of the SMP (Fig. 6). A similar finding was described in the neonatal mouse pre-Bötzinger complex, where some neurons vary their level of participation in the respiratory rhythm, from firing unfaithfully (skipping cycles) to faithfully firing on every cycle with increases in CO_2_ or decreases in pH [38]. Interestingly, while the *T. diomedea* unreliably bursting neurons can vary their level of participation in the swim network, we didn’t observe these neurons to switch the manner in which they were semi-committed to the motor program. For example, neurons didn’t switch from joining the motor program late to leaving it early. Therefore the variably bursting neurons in *T. diomedea* may be “hard-wired” with respect to the manner in which they are semi-committed to the swim network.

The finding that variably bursting neurons make up a small but significant proportion of the bursting neurons in the *T. diomedea* swim network underscores the power of VSD imaging. Previous researchers, including ourselves, may have recorded intracellularly from such unreliably bursting neurons in *T. diomedea*, but without simultaneously being aware of the larger-scale network activity, their variable participation in the motor program may not have been apparent, nor would one have been able to quantify the number of neurons that burst in such an unreliable fashion. Indeed, examples of unreliably bursting neurons can be found in the *Aplysia* literature [39], [40]. However, in these papers, the unreliably bursting nature of these neurons is not commented upon.

An advantage of examining simpler nervous systems with fewer neurons, many of them identified, is that we are now in a position explore the cellular mechanisms underlying the unreliable neuronal participation we have identified in this study. For example, future studies using sharp electrodes could map out the connections between variably participating neurons and members of the *T. diomedea* swim CPG (see **Fig. 8**).

Our present findings contribute to a growing body of evidence supporting the variable and fluid nature of rhythmic motor networks. Motor networks may be comprised of both hard-wired and variably participating neurons, the latter which can wander in and out of the network during normal operation. What might be the advantage of such shifting coalitions of participating neurons? First, flexible network structures could facilitate memory storage. In preliminary studies we have found that semi-committed neurons become fully committed to the *T. diomedea* swim network with sensitization, a non-associative form of learning [41]. Next, it is well known that cortical circuits are able to functionally reorganize following stroke or peripheral nerve injury to regain function [42]–[44]. Variably-committed neurons could serve as reserve pools that can be recruited into circuits to bolster the function of damaged networks. In summary, our present finding of variably bursting neurons in networks mediating highly stereotypic behaviors in three different species is consistent with the idea that brain networks may be more fluid in their moment-to-moment structure than has been generally appreciated.

## Methods

### Preparation

*Tritonia diomedea* and *Tritonia festiva* central ganglia consisting of the bilaterally symmetric cerebral, pleural and pedal ganglia, and *Aplysia californica* central ganglia consisting of the buccal, cerebral, pleural and pedal ganglia, were dissected out and pinned onto the bottom of a Sylgard (Dow Corning) lined Petri dish containing Instant Ocean artificial seawater (Aquarium Systems). The thick connective tissue covering the ganglia and nerves was removed with fine forceps and scissors (for intracellular recording experiments the thin protective sheath covering the neurons was also removed). The preparation was then transferred and pinned to the Sylgard-lined coverslip bottom of the recording chamber used for optical recording (PC-H perfusion chamber, Siskiyou). In many experiments, to increase the number of neurons in focus the ganglion to be imaged was flattened somewhat by pressing a cover slip fragment down upon it that was held in place with small blobs of Vaseline placed on the recording chamber floor.

### Optical Recording

Imaging was performed with an Olympus BX51WI microscope equipped with either 10×0.6NA or 20×0.95NA water immersion objectives. Preparation temperature was maintained at 10–11°C for *Tritonia* and 16–17°C for *Aplysia*, using Instant Ocean passed through a feedback-controlled in-line Peltier cooling system (Model SC-20, Warner Instruments). Temperature was monitored with a BAT-12 thermometer fitted with an IT-18 microprobe (Physitemp, Inc) positioned near the ganglion being imaged. For staining, the room was darkened and the perfusion saline was switched to saline containing the fast voltage sensitive absorbance dye RH-155 (Anaspec). Staining was carried out in one of two ways: either 5 min of 0.3 mg/ml or 1.5 hr of 0.03 mg/ml RH-155 in saline. In all cases the preparation was then perfused with 0.03 mg/ml RH-155 throughout the experiment. Trans-illumination was provided with light from a 100 W tungsten halogen lamphouse that was first passed through an electronic shutter (Model VS35 Vincent Associates), a 725/25 bandpass filter (Chroma Technology), and a 0.9 NA flip top achromat Nikon condenser on its way to the preparation. 100% of the light from the objective was directed either to an Optronics Microfire digital camera used for focusing and to obtain an image of the preparation to superimpose with the imaging data, or to the parfocal focusing surface of a 464-element photodiode array (NeuroPDA-III, RedShirtImaging) sampled at 1600 Hz. In the two *Tritonia* species, rhythmic swim motor programs were elicited using 1 to 2 s, 10 V, 10 Hz trains of 2 ms monophasic pulses delivered via suction electrode to pedal nerve 3. In *A. californica* rhythmic crawling motor programs were elicited by 120 to 155 s, 4 to 10 V, 1 Hz trains of 2 ms monophasic pulses delivered via suction electrode to pedal nerve 9.

### Sharp Electrode Recordings

Intracellular recordings were obtained with 15–30 MΩ electrodes filled with 3 M KCl or 3 M K-acetate connected to a Dagan IX2-700 dual intracellular amplifier. The resulting signals were digitized at 2 KHz with a BioPac MP 150 data acquisition system.

### Data Analysis

Optical data were bandpass filtered in software (5 Hz high pass and 100 Hz low pass Butterworth filters), and then spike-sorted with ICA in MATLAB to yield single neuron action potential traces (independent components). The accuracy of ICA was recently validated by us using simultaneous intracellular and optical recordings from neurons in various central ganglia of *T. diomedea* and *A. californica* [29].

### Identification of Semi-committed Neurons

The following procedure, implemented in MATLAB, was used to objectively identify neurons semi-committed to the swim motor program. All post-ICA independent component traces that burst in phase with at least one cycle of the swim rhythm were deemed swim network neurons and a user-specified threshold was applied to generate a binary spike time trace for each one, with spike peaks represented as ones and all other time points as zeros. Selected, reliably firing neurons (firing bursts on every cycle of the motor program) were then used to establish two templates for expected burst times, one for dorsal phase, and one for ventral phase neurons, after which each neuron was assigned by the program to one or the other group. The program then examined each neuron and identified it as semi-committed to the motor program if its firing in any template burst window fell below 1/10^th^ of that neuron’s strongest burst.
